# Seeing Beyond the Microscope: Artificial Intelligence and Fluorescence Confocal Digital Imaging in Pediatric Surgical Pathology

**DOI:** 10.3390/children12121608

**Published:** 2025-11-26

**Authors:** Donatella Di Fabrizio, Gloria Daziani, Ilir Qose, Edoardo Bindi, Michele Ilari, Alessandra Filosa, Francesco Paolo Busardò, Gaia Goteri, Giovanni Cobellis

**Affiliations:** 1 Pediatric Surgery Unit, Salesi Children’s Hospital, Polytechnic University of Marche, 60123 Ancona, Italy; 2 Section of Legal Medicine, Department of Biomedical Sciences and Public Health, Polytechnic University of Marche, 60126 Ancona, Italy; 3 Department of Biomedical Sciences and Public Health, Institute of Pathological Anatomy, Polytechnic University of Marche, 60126 Ancona, Italy; 4 Department of Specialized Clinical and Odontostomatological Sciences, Polytechnic University of Marche, 60126 Ancona, Italy

**Keywords:** ex vivo fluorescence confocal microscopy, digital pathology, children, artificial intelligence, large language models

## Abstract

**Highlights:**

**What are the main findings?**

• Integration of DP, FCM, and LLMs achieved high diagnostic accuracy in pediatric surgical pathology.
• Adding IHC data improved AI performance, resolving false negatives and reaching 100% sensitivity in neoplastic cases.

**What is the implication of the main finding?**

• AI-assisted DP workflows can enhance diagnostic efficiency and intraoperative decision-making.
• These results highlight the potential of AI to complement expert pathologists and accelerate the adoption of digital workflows in pediatric pathology.

**Abstract:**

**Background**: Digital pathology (DP) combined with fluorescence confocal microscopy (FCM) allows rapid tissue assessment while preserving specimens. Artificial intelligence (AI) and large language models (LLMs) may enhance diagnostic workflows, but their role in pediatric surgical pathology is largely unexplored. **Methods:** We conducted a prospective, single-center study including 20 pediatric surgical cases with ex vivo FCM images acquired intraoperatively. Two commercially available LLMs, GPT-4V (AnPathology-Gpt) and Claude 3.7 Sonnet (AnPathology Project), were tested using structured prompts to generate diagnostic reports with and without immunohistochemistry (IHC) data, when available. Outputs were compared against the gold standard diagnosis by an experienced pediatric pathologist. Diagnostic performance was evaluated through accuracy, sensitivity, specificity, and Cohen’s kappa. A paired sub-analysis was performed for cases with IHC (*n* = 5), and a sensitivity analysis excluding IHC cases (*n* = 15) was conducted. **Results:** Across all 20 cases, AnPathology-Gpt achieved 85% accuracy, 100% sensitivity, 86% specificity, and κ = 0.78, while AnPathology Project reached 80% accuracy, 100% sensitivity, 57% specificity, and κ = 0.63. Both models correctly identified all 13 neoplastic cases, with errors limited to non-neoplastic lesions mimicking tumors. In the IHC sub-analysis, accuracy improved from 40% to 80% and sensitivity from 50% to 100% for both models, resolving two false negatives observed in the FCM-only evaluation. Sensitivity analysis excluding IHC confirmed consistency of the results. **Conclusions:** This pilot study demonstrates that multimodal LLMs can support accurate and rapid diagnosis in pediatric digital pathology. The addition of IHC improves performance in diagnostically complex cases. Larger multicenter studies are needed to validate these findings and to define the role of AI-assisted workflows in pediatric surgical pathology.

## 1. Introduction

According to the European Society of Digital and Integrative Pathology (ESDIP), Digital Pathology (DP) represents a comprehensive approach that involves intervention at every step of the workflow in pathology laboratories, starting and encouraging innovation [1].

Building on this concept, DP has progressively evolved from the simple digitalization of histological slides to a fully integrated digital ecosystem. Digitalization represents the foundation of DP, enabling the acquisition, storage, and remote visualization of whole-slide images; however, contemporary DP now extends to advanced image management platforms, telepathology, and computational image analysis tools [2,3]. This paradigm shift reflects a transition from digitizing glass slides to re-engineering the entire diagnostic workflow through data integration and technological innovation [1]. Within this expanded digital framework, artificial intelligence (AI) has emerged as a key driver of innovation, complementing DP by enabling automated tissue analysis, quantitative feature extraction, and enhanced diagnostic support [4]. In 2017 the Food and Drug Administration (FDA) granted the first approval for WSI use in primary diagnosis and, for the first time, the application of DP for clinical purposes was legitimated [5].

Alongside the increasing use of DP, the advent of new analytical techniques such as Fluorescent Confocal Microscopy (FCM) has further supported its development, offering several advantages across different clinical fields, such as the preservation of specimens, as it does not require traditional staining—a feature particularly relevant in pediatric surgery [6,7]. After preliminary analysis with FCM, the same surgical specimen can still be used for further molecular investigations, as demonstrated by Gretser et al. in the evaluation and isolation of tumor cells from rare pediatric tumors [8].

When used ex vivo, FCM significantly reduces processing and analysis time to approximately 5 min, compared to the 24–48 h typically required for standard pathological evaluation of biopsies and surgical specimens [6,7,9,10]. This substantial time saving translates into a meaningful reduction in anesthesia duration, which is especially critical in pediatric patients [6,7].

In this context, AI can enhance DP by processing large volumes of complex data, a key advantage in pediatrics, where rare diseases and the need for fast, accurate diagnoses are common [11]. AI can detect subtle patterns and extract quantitative features often missed by the human eye, improving diagnostic sensitivity [12]. It thus serves as a valuable tool to support and refine pathologists’ assessments [13].

The aim of this study was to explore the potential of integrating DP, through the use of FCM, within an AI model, to support the diagnostic accuracy in pediatric surgical pathology.

## 2. Materials and Methods

### 2.1. Study Design

This was a prospective, single-center observational study conducted at the Pediatric Surgery Unit of the Salesi Children’s Hospital and the Department of Pathological Anatomy, Polytechnic University of Marche, Ancona, Italy.

Twenty consecutive pediatric patients undergoing surgical procedures between November 2023 and March 2025 were included. Cases were selected based on the availability of high-quality ex vivo FCM images acquired intraoperatively, the presence of a definitive histopathological diagnosis established by an experienced pediatric pathologist serving as the gold standard, and the completeness of associated clinical and imaging metadata. Cases were excluded when the gold standard diagnosis was unavailable, image quality was insufficient, or essential diagnostic information was missing. All images and metadata were anonymized (sequentially coded from ID01 to ID20) before AI processing, in accordance with institutional and GDPR pediatric data protection standards. No identifiable patient information was uploaded to external systems.

### 2.2. Ex Vivo Fluorescence Confocal Microscopy

Ex vivo fluorescence confocal microscopy was performed using the VivaScope 2500 M-G4 system (Mavig GmbH, Munich, Germany; Caliber I.D., Rochester, NY, USA). The device operates with dual-laser excitation at 488 nm and 785 nm, producing grayscale reflectance and fluorescence images at a resolution of 1024 × 1024 pixels per tile [6,14]. Individual tiles were automatically stitched into high-resolution mosaics and digitally pseudo-colored to reproduce the appearance of conventional hematoxylin and eosin (H&E) staining. For each patient, between one and three FCM images were collected from biopsy or surgical specimens. Among cases with available immunohistochemistry (IHC), a random subset (*n* = 5) was analyzed to assess whether combining FCM with IHC data could influence AI diagnostic performance.

### 2.3. AI Models and Prompt Engineering

Two commercially available LLM-based AI diagnostic models were evaluated: AnPathology-Gpt, based on GPT-4V (OpenAI, Plus plan), and AnPathology Project, based on Claude 3.7 Sonnet (Anthropic, Professional plan). Both models were configured using institution-specific prompt engineering to standardize diagnostic reasoning and reporting. Structured prompts were designed to ensure consistency in terminology and workflow, instructing the models to analyze multimodal inputs, including FCM images, IHC results when available, clinical metadata, and laboratory findings (Supplementary Materials). The models were asked to provide a structured primary diagnosis, a ranked list of differential diagnoses with estimated probabilities, and recommendations for additional diagnostic investigations when appropriate. To ensure reproducibility, identical data inputs were provided to both models for each case, and analyses were performed both with and without IHC integration when IHC was available.

For every case, AI-generated diagnostic outputs were compared against the gold standard established by a senior pediatric pathologist with over 15 years of experience. A diagnosis was considered correct when the primary diagnosis proposed by the AI matched the gold standard. When models returned a differential diagnosis list rather than a single categorical output, the prediction was considered correct only if the gold-standard diagnosis was either the primary AI diagnosis or, if listed among differential diagnoses, ranked first with a confidence score ≥50%. Lower-ranked or low-confidence suggestions were not considered correct. Based on the gold standard, cases were classified as neoplastic or non-neoplastic to evaluate the models’ ability to discriminate between tumoral and non-tumoral lesions.

The primary analysis followed an intention-to-diagnose approach and included all 20 cases.

### 2.4. Statistical Analysis

Diagnostic performance metrics calculated for each LLM included accuracy, sensitivity, specificity, and Cohen’s kappa coefficient to quantify agreement beyond chance. True positives (TP) were defined as neoplastic cases correctly identified, true negatives (TN) as benign cases correctly classified, false positives (FP) as benign cases incorrectly labeled as neoplastic, and false negatives (FN) as neoplastic cases missed by the AI. Subgroup analyses were performed separately for neoplastic and non-neoplastic lesions.

A predefined paired sub-analysis was performed among cases with available IHC (*n* = 5) to evaluate the impact of adding IHC information to the FCM-based diagnostic workflow. For each LLM, diagnostic performance with FCM alone was compared with performance after adding IHC (FCM + IHC) within the same cases. The primary endpoints of this sub-analysis were overall accuracy and sensitivity, while specificity among non-neoplastic cases was evaluated when applicable. Paired binary outcomes (correct vs. incorrect primary diagnosis) were compared using McNemar’s exact test, and risk differences were estimated with non-parametric bootstrap resampling (5000 iterations) to calculate 95% confidence intervals. Given the limited sample size, this analysis was considered exploratory.

Finally, a sensitivity analysis was conducted by repeating the primary performance comparison after excluding cases with available IHC (*n* = 15) to confirm the robustness of the findings.

## 3. Results

Twenty pediatric cases were analyzed, each with a definitive gold-standard diagnosis established by the pathologist. Based on the gold standard, 13 cases were classified as neoplastic, including both malignant and benign neoplasms, and 7 were classified as non-neoplastic, encompassing reactive, inflammatory, or reparative lesions. Both AI systems processed the same multimodal inputs for each case.

### 3.1. Global Diagnostic Performance (Primary Analysis) (Table 1)

When diagnostic performance was evaluated across all 20 cases, AnPathology-Gpt correctly identified 13 of 13 neoplastic lesions and 6 of 7 non-neoplastic lesions, resulting in one false positive and no false negatives. AnPathology Project also correctly identified all neoplastic lesions but classified only 4 of 7 non-neoplastic lesions correctly, resulting in three false positives and no false negatives (Table 1).

**Table 1:** Case-level comparison between gold standard diagnoses and AI-generated outputs across 20 pediatric surgical cases. For each case, the gold standard diagnosis and classification (tumor vs. non-tumor) are reported alongside the predictions of AnPathology-Gpt (GPT-4V) and AnPathology Project (Claude 3.7). Classifications are indicated as true positive (TP), true negative (TN), false positive (FP), or false negative (FN). Misclassifications occurred exclusively in non-tumoral cases mimicking neoplasia. For cases with available immunohistochemistry (IHC), this table reports the final diagnostic output after integration of both FCM and IHC results.

ID	Gold Standard Diagnosis	Tumor or Non-Tumor	AnPathology-Gpt Diagnosis	GPT Classification	AnPathology Project Diagnosis	Claude Classification
1	Atypical Spitz nevus	Tumor	Atypical Spitz tumor	TP	Spitz nevus/dd melanoma	FN
2	Wilms tumor	Tumor	Wilms tumor	TP	Wilms tumor	TP
3	Hyperplastic lymphadenopathy	Non-tumor	Reactive lymphoid hyperplasia	TN	Granulomatous lymphadenitis	TN
4	Malignant germ cell neoplasm	Tumor	Seminoma/Dysgerminoma	TP	Germ cell tumor	TP
5	Hodgkin lymphoma	Tumor	Classical Hodgkin lymphoma	TP	EBV-associated Hodgkin lymphoma	TP
6	Embryonal hepatic sarcoma	Tumor	Embryonal hepatic sarcoma	TP	Undifferentiated embryonal sarcoma	TP
7	Rhabdomyosarcoma	Tumor	Alveolar rhabdomyosarcoma	TP	Rhabdomyosarcoma	TP
8	Neuroblastoma	Tumor	Neuroblastoma	TP	Neuroblastoma	TP
9	Florid follicular hyperplasia	Non-tumor	Florid follicular hyperplasia	TN	Florid follicular hyperplasia	TN
10	Poorly differentiated neuroblastoma	Tumor	Neuroblastoma	TP	Neuroblastoma	TP
11	Cholestatic hepatopathy	Non-tumor	Neonatal hepatitis	TN	PFIC/bile acid defect	TN
12	Thymic hyperplasia	Non-tumor	Thymoma/T-LBL	FP	Precursor T-LBL lymphoma	FP
13	Metanephric adenoma	Tumor	Metanephric adenoma	TP	Metanephric adenoma	TP
14	Benign reparative lesion	Non-tumor	Osteomyelitis/histiocytosis	TN	Ewing sarcoma (85%)	FP
15	Benign aneurysmal bone cyst	Non-tumor	Aneurysmal bone cyst	TN	Langerhans cell histiocytosis	FP
16	Benign reactive lesion	Non-tumor	Fibro-osseous benign lesion	TN	Fibro-osseous benign lesion	TN
17	Inflammatory benign lesion	Non-tumor	Chronic osteomyelitis	TN	Pigmented villonodular synovitis	FP
18	Ewing sarcoma	Tumor	Ewing sarcoma	TP	Ewing sarcoma	TP
19	Pilocytic astrocytoma	Tumor	Pilocytic astrocytoma	TP	Pilocytic astrocytoma	TP
20	Choroid plexus papilloma (G1)	Tumor	Residual papilloma	TP	Residual papilloma	TP

For AnPathology-Gpt, the overall accuracy was 85%, sensitivity 100%, specificity 86%, and Cohen’s κ 0.78, indicating substantial agreement with the gold standard. For AnPathology Project, the overall accuracy was 80%, sensitivity 100%, specificity 57%, and κ 0.63, consistent with moderate agreement (Table 2).

No false negatives were observed for either model. All misclassifications occurred in non-neoplastic lesions mimicking neoplasia, such as thymic hyperplasia, benign bone lesions, and reactive lymphoid proliferations.

### 3.2. Paired Sub-Analysis: Impact of IHC (N = 5) (Table 3)

Among the five cases in which IHC data were available, diagnostic performance was compared within the same cases and models using FCM-only versus FCM + IHC inputs. Without IHC, both models achieved an accuracy of 40%, correctly classifying only two of the five cases. Two neoplastic lesions were misclassified as non-neoplastic by both models in the FCM-only condition. After adding IHC, accuracy improved to 80% for both models, with all neoplastic lesions correctly classified and only one benign lesion (florid follicular hyperplasia, ID09) consistently recognized as non-neoplastic.

Sensitivity improved markedly from 50% to 100% with the addition of IHC, while specificity remained unchanged because only one benign case was present in this subset. McNemar’s exact test confirmed a statistically significant improvement in classification accuracy for both models after adding IHC (*p* < 0.05).

The detailed paired results for each case are presented in Table 3. Cases ID01 (atypical Spitz nevus) and ID06 (embryonal hepatic sarcoma) highlight the clinical utility of IHC integration: both were misclassified without IHC but correctly diagnosed after IHC, suggesting that immunophenotypic data can be critical in resolving ambiguous pediatric lesions.

**Table 3:** Paired sub-analysis of the five cases with available immunohistochemistry (IHC). Diagnostic outputs of AnPathology-Gpt (GPT-4V) and AnPathology Project (Claude 3.7) are shown with FCM-only data and after the addition of IHC (FCM + IHC). Both models improved accuracy and sensitivity when IHC was integrated, resolving two false negatives and reaching 100% sensitivity in neoplastic cases.

ID	Gold Standard Diagnosis	Gold (Tumor?)	AnPathology-Gpt FCM-Only	AnPathology-Gpt FCM + IHC	AnPathology Project FCM-Only	AnPathology Project FCM + IHC
1	Atypical Spitz nevus	Tumor	Classic Spitz nevus (FN)	Atypical Spitz tumor (TP)	Spitz nevus (FN)	Atypical Spitz tumor (TP)
5	Hodgkin lymphoma	Tumor	Nodular sclerosis HL (TP)	Classical HL nodular sclerosis (TP)	HL nodular sclerosis (TP)	EBV-associated HL (TP)
6	Embryonal hepatic sarcoma	Tumor	Likely hepatoblastoma (FN)	Embryonal hepatic sarcoma (TP)	Mixed hepatoblastoma (FN)	Undifferentiated embryonal sarcoma (TP)
9	Florid follicular hyperplasia	Non-tumor	Reactive hyperplasia (TN)	Florid hyperplasia (TN)	Reactive hyperplasia (TN)	Florid hyperplasia (TN)
20	Choroid plexus papilloma (G1)	Tumor	Residual papilloma (TP)	Residual papilloma (TP)	Residual papilloma (TP)	Residual papilloma (TP)

### 3.3. Sensitivity Analysis Excluding IHC Cases (N = 15)

To assess the robustness of the primary results, we repeated the analysis after excluding the five cases with available IHC. Among the remaining 15 cases, AnPathology-Gpt achieved an accuracy of 87% (13/15 correct classifications), sensitivity 100%, and specificity 71%, while AnPathology Project achieved an accuracy of 80% (12/15 correct classifications), sensitivity 100%, and specificity 57%. These findings were consistent with the primary analysis, confirming that the global results were not driven by the subset of cases where IHC was available.

## 4. Discussion

To the best of our knowledge, this represents the first study exploring the potential for integration between DP, FCM, and AI in the field of pediatric surgical pathology.

Digital pathology has already emerged as a valid alternative to conventional laboratory protocols, enabling faster turnaround times and new opportunities for workflow optimization [15]. When combined with advanced technologies such as FCM, DP can further enhance diagnostic efficiency and accuracy, with clear benefits for patients [6,11,16]. In this early-phase pilot setting, our objective was not to propose an alternative to standard pathological workflows, but to explore whether the combined use of FCM and AI could complement routine practice and support timely diagnostic assessment.

The application of AI models in pathological fields demonstrated that diagnoses gained through AI could reach the precision of expert pathologists’ diagnoses and sometimes surpass them [17]. In particular, in the study conducted by Strotzer et al., GPT-4V proved to be promising in interpreting radiological images, adapting to field-specific requirements even if it could not interpret medical images well [18]. For instance, use of AI with “clinical profile” prompts resulted in a valid support in ophthalmic diagnoses [19]. Although it showed high levels of inaccuracy in given image interpretation, other study demonstrated that GPT-4V showed potential in radiological image interpretation [20,21,22].

In this study, we evaluated GPT-4V (AnPathology-Gpt) and Claude 3.7 Sonnet (AnPathology Project), two of the most widely used commercially available LLMs. GPT-4V, based on the GPT-4-turbo architecture, has the ability to process both text and images, supporting advanced visual analysis and data interpretation. Claude 3.7 Sonnet also demonstrated the ability to analyze complex images and to integrate language, vision, and reasoning. At the time of this study, both represented the most recent and high-performing versions of their respective platforms.

Initially, analysis was performed using both open-ended and structured prompts. After checking the first results, the project proceeded with structured prompts only. In fact, the importance of using a structured prompt to guide AI in data interpretation is already well established [23], and only results obtained from structured prompt could be suitable in a clinical and professional context [24]. Use of appropriate prompt is necessary to guide AI during diagnosis development [25].

Both LLMs demonstrated excellent performance in identifying pediatric neoplasms, achieving a sensitivity of 100% with no false negatives. AnPathology-Gpt showed higher specificity than AnPathology Project (86% vs. 57%), resulting in fewer false positives and a better overall agreement with the gold standard. The integration of immunohistochemistry (IHC) improved diagnostic performance in the five complex cases where it was available, resolving two false negatives present in the FCM-only analysis. In these challenging cases, IHC increased accuracy from 40% to 80% and sensitivity from 50% to 100%.

These findings support the potential role of multimodal LLMs as decision-support tools in pediatric digital pathology, particularly during intraoperative consultations when rapid diagnosis is critical. However, the higher rate of false positives observed with AnPathology Project highlights the need for controlled implementation and continued reliance on expert pathologists.

The improved performance observed when integrating multimodal data, including IHC, suggests that an AI-assisted workflow incorporating complementary information could enhance diagnostic accuracy in complex cases.

The addition of IHC data had a marked effect in resolving diagnostically challenging cases. Two illustrative examples highlight its impact. In case ID01 (atypical Spitz nevus), both models misclassified the lesion as a benign Spitz nevus without IHC but correctly identified it as an atypical Spitz tumor once IHC results were provided. Similarly, in case ID06 (embryonal hepatic sarcoma), both models initially classified the lesion as hepatoblastoma but corrected their diagnosis after integrating IHC data. These findings suggest that IHC provides critical discriminative information in borderline and ambiguous pediatric lesions and enhances the reliability of LLM-assisted workflows. Future developments may further strengthen performance by integrating additional pathological, radiological, and clinical inputs, particularly in rare pediatric diseases where multimodal data are often decisive.

The study has several limitations. The sample size was small, limiting the statistical power and generalizability of the findings. One of the election criteria for inclusion of cases was the adequate digital data availability, which may have led to the choice of cases that were easier to interpret or exhibited clear diagnostic features. It was not possible to insert DICOM data (CT scan and MRI) due to their size, but they could be integrated after a preliminary screening to select the most significant ones. Furthermore, the dataset included only surgically resected lesions, meaning that the absence of normal or unequivocally negative controls could overestimate sensitivity. The diagnostic prompts used for the models were optimized for this institution, which may limit reproducibility in other settings. Finally, LLMs evolve rapidly, and future models are likely to outperform the versions evaluated here. Future studies should include multicenter datasets and harmonized acquisition protocols to support model standardization, reproducibility, and broader clinical translation in pediatric pathology.

## 5. Conclusions

The combined use of DP, FCM and AI may contribute to more efficient diagnostic pathways in pediatric surgical pathology, where FCM enables rapid intraoperative tissue assessment and AI currently serves as a decision-support tool to assist interpretation. This pilot study demonstrated that large language models such as GPT-4V and Claude 3.7 Sonnet can generate reliable diagnostic outputs when provided with appropriate clinical context and structured information. Although the results are promising, the limited sample size and heterogeneous case mix underline the need for further research.

## Figures and Tables

**Table 2:** Global diagnostic performance of AnPathology-Gpt (GPT-4V) and AnPathology Project (Claude 3.7) across all 20 pediatric cases. Reported metrics include the number of true positives (TP), true negatives (TN), false positives (FP), and false negatives (FN), as well as overall accuracy, sensitivity, specificity, and Cohen’s kappa coefficient (κ). AnPathology-Gpt demonstrated higher specificity and agreement with the gold standard compared to AnPathology Project.

Metric	AnPathology-Gpt (GPT-4V)	AnPathology Project (Claude 3.7)
True Positives (TP)	13	13
True Negatives (TN)	6	4
False Positives (FP)	1	3
False Negatives (FN)	0	0
Accuracy	85%	80%
Sensitivity	100%	100%
Specificity	86%	57%
Cohen’s κ	0.78	0.63

## Data Availability

The original contributions presented in this study are included in the article/Supplementary Materials. Further inquiries can be directed to the corresponding authors.

## Supplementary Materials

The following supporting information can be downloaded at: https://www.mdpi.com/article/10.3390/children12121608/s1.
