# Supplementary material for: Seeing Beyond the Microscope: Artificial Intelligence and Fluorescence Confocal Digital Imaging in Pediatric Surgical Pathology
Source: Children (Basel). 2025 Nov 26;12(12):1608. doi: 10.3390/children12121608 (PMC12731743; doi:10.3390/children12121608)
Supplement: Supplementary file 1 [file children-12-01608-s001.zip › children-3938697-supplementary.pdf]

a. AI Configuration and analyses execution

Starting from professional subscription to ChatGPT Plus (for GPT-4V) and Professional Plan (for Claude 3.7 Sonnet, Anthropic), structured prompts were developed and named AnPathology-Gpt and AnPathology Project, respectively.
Information regarding the structured prompt details is depicted in Supplementary Table S1.
Analyses were performed using AnPathology-Gpt and AnPathology Project, both with (advanced analysis) and without (preliminary analysis) considering immunohistochemical (IHC) data (when available), to compare results.

b. Commercial LLM Selection

The used AI models were two commercially available ML models. In particular we used professional subscription to ChatGPT Plus (for GPT-4V) (cost: €20/month) and professional Plan for Claude 3.7 Sonnet (Anthropic) (cost: €20/month).

c. Prompt Engineering Development

Structured prompts were developed and optimized through iterative testing, incorporating institutional-specific knowledge and terminology.
Both AI systems were enhanced with Academic Foundation which includes complete pathology lecture notes from the University's Medicine program, ensuring use of institution-specific terminology and diagnostic approaches; Clinical Templates consistent in official report templates and forms from the UnivPM Pathological Anatomy Clinic; Institutional Language with specific Italian-to-English medical terminology mappings used at University; and Diagnostic Workflows based on step-by-step diagnostic protocols following University's established procedures.

d. Prompt engineering approach

The prompt engineering approach was focused on: structured clinical information presentation matching University's case presentation format, clear diagnostic query formulation using institutional terminology, integration of multimodal data (FCM images, clinical data, IHC when available),
standardized output format requirements following University’s reporting templates
and responses formatted to match the communication style familiar to University’s clinic staff.
The prompt submitted to the AI system was:
“You are an advanced diagnostic assistant specializing in digital pediatric pathology, designed to analyze and integrate multimodal data (histology, radiology, laboratory) to support accurate pediatric diagnoses. It is important that you focus primarily on the pathological analysis and reporting of biopsy material, such as biopsies or confocal images. Your work will then be compared with the report made in the laboratory by the pathologist. Use other data only if necessary and if it can help you with your pathological diagnosis.”

e. AI Output examples (ID14)

Available data for case ID14 were two CM images and some clinical information (lameness, osteolytic lesion of left talus). IHC images, diagnostic images, clinical data or diagnostic report were not present. Moreover, biopsy site, type of tissue and patient age are listed.

The gold standard diagnosis was “Benign bone lesion, most likely of reactive nature”.

AnPathology-Gpt

I. *Color normalization and contrast* enhancement were applied to better highlight the tissue structures and any potential anomalies (Figure 1).

II. *Adaptive thresholding* was used to segment the tissue and highlight:
Cellular density, Bone structure and lacunae, Areas of tissue disorganization. In the images: The white areas represent the cellular and bone structures. The black areas highlight the cavities or possible areas of necrosis (Figure 2).

III. *The tissue contours were delineated and accentuated on enhanced images.* The green lines demarcate cellular structures and tissue margins. The denser regions may suggest cellular accumulation, inflammatory infiltrate, or morphological alterations (Figure 3).

IV. Necrosis was quantified in both samples, with the following results (Figure 4):
Biopsy 16A → 35.35% necrotic area,
Biopsy 16B → 20.82% necrotic area.

V. The areas of inflammatory infiltrate were highlighted, appearing in red in (Figure 5) section, indicating an intense inflammatory response. Sample 16B: The infiltrate remains present but to a slightly lesser extent compared to sample 16A.

VI. Cell nuclei were highlighted in yellow (Figure 6), and cellular density was calculated per million pixels:
Sample 16A → Density: 160.91 nuclei/million px,
Sample 16B → Density: 200.64 nuclei/million px.
Interpretation of Results: Sample 16B shows higher nuclear density, which may suggest active cell proliferation or a denser inflammatory infiltrate. Sample 16A displays lower nuclear density, potentially correlating with a more extensive necrotic area.

Diagnosis: The case with the greatest similarity is Osteomyelitis, followed by Histiocytosis and Reactive Inflammation (Gold standard Report AP: Benign bone lesion, most likely of reactive nature).

AnPathology Project

I. *Preliminary Evaluation of the Images*
The images show sections of bone tissue obtained through confocal microscopy, stained with Hematoxylin and Eosin (H&E), from a 1-year-old male patient with persistent lameness for 3 months and osteolytic lesions in the left talus identified by imaging (X-ray, CT scan, MRI).

II. *Morphological Analysis of the Tissue*
Image 1: Altered bone architecture is observed. White areas (osteolytic) with a clear loss of normal bone structure. Dense cellular infiltrate with dark purple coloration in focal areas. Disorganized growth pattern with bone marrow infiltration. Zones of

destruction of the normal bone matrix. *Image 2:* Magnification showing cells in greater detail. Presence of relatively monomorphic cells with hyperchromatic nuclei. Supporting fibrovascular stroma. Infiltrative distribution of pathological cells. Sparse or absent normal bone tissue. *3. Cytological Features:* Small to medium-sized cells with round-oval, intensely basophilic nuclei. Finely dispersed chromatin. Sparse cytoplasm. High nuclear/cytoplasmic ratio. Diffuse growth pattern with areas of aggregation. Presence of some mitotic figures. Absence of reactive bone formation. *4. Correlation with Clinical and Radiological Data:* The histological pattern, correlated with: Patient's age (1 year), Clinical presentation (lameness for 3 months), Lesion site (left talus), Radiological features (osteolytic lesions), suggests a high-grade primitive mesenchymal neoplasm with aggressive behavior.

Differential Diagnosis: Considering all available data, the main differential diagnoses include Ewing's Sarcoma/PNET (Primitive Neuroectodermal Tumor) – 85%, Small Cell Osteosarcoma – 40%, Primary Bone Lymphoma – 30%, Langerhans Cell Histiocytosis – 25%.

All other cases results are depicted in Supplementary Table S2 in comparison with the gold standard.

AnPathology: structured prompt
**A. Definition and contest**
1. Specific clinical assessment
2. Medical history
3. Pathology under examination
**B. Supported input**
1. Digital microscopic slides<br>- SVS, NDPL, SCN, TIFF (>20x)
2. Clinical Record<br>Age, gender, symptoms, family history<br>Medical history, blood test results, previous reports
3. Radiological imaging<br>CT scan, MRI, PET, ultrasounds<br>Date, type of scan, anatomical region
4. Molecular/immunohistochemical data<br>IHC panels, specific markers<br>Genetic mutations, expression profiles
**C. Analytical protocol**
1. Histopathology<br>Preprocessing: color normalization, tissue segmentation, hotspot detection<br>Morphological characterization: tissue architecture, cellular morphology, necrotic index, necrotic area, vascular invasion<br>Comparison with pediatric database: comparison with age-specific digital atlases, similarity analysis with previous cases (3-10)
2. Clinical data analysis<br>Automatic extraction of altered biochemical markers, temporal evolution of symptoms, relevant family history<br>Correlation of demographic data: disease incidence by age group, values interpreted according to pediatric ranges
3. Imaging analysis<br>Lesional characterization: precise 3D localization, dimensions, margins, perfusion pattern, relationship with adjacent anatomical structures<br>Multimodal integration: imaging-histology co-registration, pathological spread 3D mapping
**D. Structured output**
1. Integrated report<br>Header: patient ID, date, requesting physician<br>Clinical summary and morphological analysis<br>Radiological correlation: lesion characterization<br>Differential diagnosis: top 3-5 diagnosis with probability (0-100%), supporting/contrasting factors for each<br>Scoring system: confidence, urgency (1-5)
2. Clinical recommendations<br>Additional IHC/molecular panels<br>Complementary radiological exams
3. Visual representation<br>Pathological areas histological heat map<br>Integrated radio pathological 3D reconstructions
**E. Ethical and legal considerations**
1. Preliminary nature of AI
2. GDPR compliance for pediatric data
**F. References**
1. Use of standard classifications (WHO, Pathology outlines)
2. Diagnostic standardization criteria
3. Pediatric guidelines
4. UNIVPM and Salesi Terminology

Supplementary Table S1: Actual prompt used for LLMs Input. Engineering prompt workflow and dif-ferent used parameters.

ID	AnPathology-Gpt	AnPathology Project	AP diagnosis
01	D.No IHC. Classic Spitz nevus<br>D. With IHC. Atypical Spitz tumor	D. No IHC. Spitz nevus<br>dd . Atypical Spitz tumor (5%), Spitzoid melanoma (<5%)<br>D. With IHC. Atypical Spitz tumor<br>dd. Conventional Spitz nevus (10%)	Atypical Spitz Nevus
02	D. Wilms Tumor	D. Wilms Tumor (Nephroblastoma)	Nephroblastoma (Wilms Tumor)
03	D. Reactive lymphoid hyperplasia	D. Non-necrotizing granulomatous lymphadenitis	Hyperplastic Lymphadenopathy
04	D. Testicular seminoma (high probability)<br>Dysgerminoma (rare form in males, 10-15%)	D. Germ cell tumor post-treatment changes<br>dd. Sex cord-stromal tumor (60%), Teratoma with predominant fibrous component (40%)	Non-Germinative Malignant Germ Cell Neoplasm
05	D. No IHC. Nodular sclerosis Hodgkin's Lymphoma, post-CHT<br>D. With IHC. Classical Hodgkin's lymphoma, nodular sclerosis subtype (with focal type 2 - BNLI), partial treatment response and EBV positivity evidence	D. No IHC. Hodgkin's lymphoma (nodular sclerosis subtype, treament response evidence)<br>D. With IHC. EBV-associated nodular sclerosis Hodgkin's lymmphoma in partial remission post-chemotherapy	Hodgkin's Lymphoma, Nodular Sclerosis Type
06	D. No IHC. Lesion compatible woth aggressive pediatric liver neoplasm, hepatoblastoma(HB)<br>dd. Hepatocellular carcinoma (HCC)/Epithelioid or mesenchymal sarcoma/Rhabdois teratoid tumor<br>D with IHC. Embryonal Hepatic Sarcoma	D. No IHC. Epithelial/mixed hepatoblastoma<br>dd. Undifferentiated small cell hepatoblastoma (80%), Undifferentiated embryonal sarcoma of the liver (70%)<br>D with IHC. Undifferentiate embyonal sarcoma of the liver (UESL)	Embryonal Hepatic Sarcoma
07	D. Alveolar rhabdomyosarcome<br>dd. Metastatic neuroblastoma (5%), Ewing sarcoma (5%)	D. Rhabdomyosarcoma in regression post-chemotherapy	Malignant Mesenchymal Neoplasm, Rhabdomyosarcoma Type
08	D. Undifferentiated neuroblastoma<br>dd. Wilms tumor (nephroblastoma, 8%), Adrenal pheochromocytoma (5%)	D. Neuroblastoma (85%)<br>dd. Wilms tumor (20%), Extraosseous Ewing sarcoma (10%), Rhabdomyosarcoma (10%), Non-Hodgkin's lymphoma (5%)	Undifferentiated Neuroblastoma
09	D. No Ihc. Reactive follicular hyperplasia<br>D. With IHC . Florid reactive Follicular Hyperplasia	D. No IHC Reactive follicular hyperplasia<br>dd. Nodular lymphocyte-predominant Hodgkin lymphoma/ Pediatric follicular lymphoma<br>D. With IHC Florid reactive Follicular Hyperplasia	Florid Follicular Hyperplasia
10	D. MS neuroblastoma with partial response to chemotherapy	D. Poorly Differentiated Neuroblastoma	Poorly Differentiated Neuroblastoma
11	D. Epatite neonatale lieve con infiammazione portale e possibile eziologia colestatica a GGT bassa	D. Deficit di sintesi degli acidi biliari (70%) /Colestasi intraepatica familiare progressiva - PFIC (65%) Epatite Neonatale (40%)	Cholestatic Hepatopathy
12	D. Thymoma (50%),<br>dd. T-lymphoblastic lymphoma (30%), Thymic hyperplasia and thymic teratoma (10%)	D. Precursor T-lymphoblastic lymphoma (T-LBL, 85%)<br>dd. Type B thymoma, lymphocyte-rich (10%), Diffuse small B-cell lymphoma (5%)	Thymic hyperplasia
13	D. Before dd request: Undifferentiated neuroblastoma<br>D . After Dd request: Nephroblastoma (80%), Metanephric adenoma (15%)	D. Before dd request: Nephroblastoma (80%), Metanephric adenoma (20%)<br>D . After Dd request : Metanephric adenoma (75%), Nephroblastoma (20%)	Metanephric Adenoma
14	D. Osteomyelitis, histiocytosis	D. Ewing sarcoma/PNET (Primitive Neuroectodermal Tumor, 85%)	Benign Repairative Lesion
15	D. Aneurysmal bone cyst (ABC)<br>dd. Langerhans cell histiocytosis (20%)	D. Langerhans Cell Histiocytosis (LCH) involving the right femur<br>dd. Aneurysmal bone cyst (ABC)	Benign Aneurysmal Bone Cyst
16	D. No IHC. Chronic osteomyelitis<br>dd. Legg-Calvé-Perthes disease (15%), Fibrous dysplasia (10%)<br>D with IHC. Inflammatory type benign fibro-osseous lesion with areas of necrosis and reactive fibrosis	D. No IHC. Microcrystalline arthropathy - juvenile gout<br>dd. Pigmented villonodular synovitis (80%), Reactive/inflammatory arthritis<br>D with IHC. Inflammatory type benign fibro-osseous lesion (95%)	Benign Reactive Lesion
17	D. Chronic osteomyelitis<br>dd. Pigmented villonodular synovitis (20%), Giant cell tumor (10%)	D. Pigmented villonodular synovitis<br>dd Juvenile idiopathic arthritis (85%)/Non-suppurative chronic osteomyelitis (60%)/Osteoid osteoma(initial phase, 40%), Giant cell granuloma (30%)	Inflammatory benign lesion
18	D. Ewing sarcoma<br>dd Chronic osteomyelitis (40%), Telangiectatic osteosarcome (30%)	D. Ewing sarcoma/PNET<br>dd. Primary bone lymphoma (10%), Rhabdomyosarcoma (5%)	Ewing's Sarcoma
19	D. Pilocytic astrocytoma (40%)<br>dd. Diffuse astrocytoma (30%), Ganglioma (15%), Ependymoma (10%)	D. Pilocytic astrocytoma<br>dd. Diffuse astrocytoma (10-15%), Ependymoma (<5%)	Pilocytic astrocytoma
20	D. No IHC. Residual choroid plexus papilloma (WHO Grade 1)<br>D. With IHC. Residual choroid plexus papilloma (WHO Grade 1)	D. No IHC. Residual choroid plexus papilloma (WHO Grade 1)<br>D. With IHC. Residual choroid plexus papilloma (WHO Grade 1)	Carotid Plexus Papilloma (WHO Grade 1)

Supplementary Table S2: Diagnoses provided after AI models data analyses and pathologist diagno-ses (gold standard). D: diagnosis, dd: differential diagnosis, AP: Anatomic pathologist, IHC: Im-munohistochemical images.

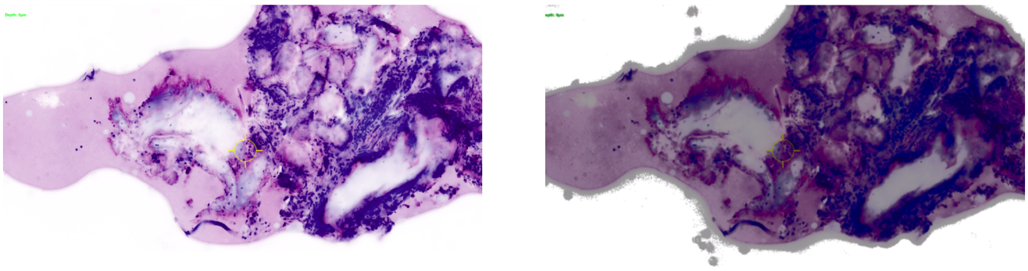

Figure S1. Case ID14. Original images (left side) and images with enhanced contrast through histogram equalization on the brightness channel (right side).

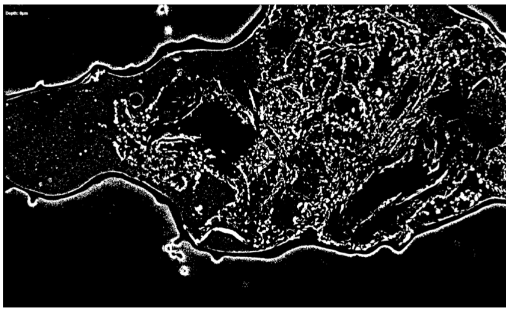

Figure S2. Case ID14. Images after segmentation process.

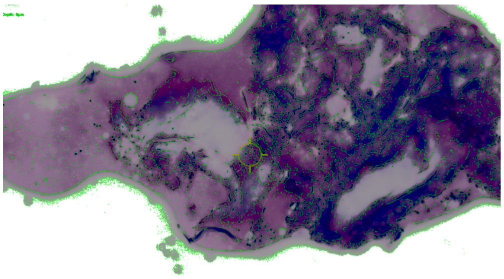

Figure S3. Case ID14. Images whit tissue contours delineated and accentuated, the green lines demarcate cellular structures and tissue margins.

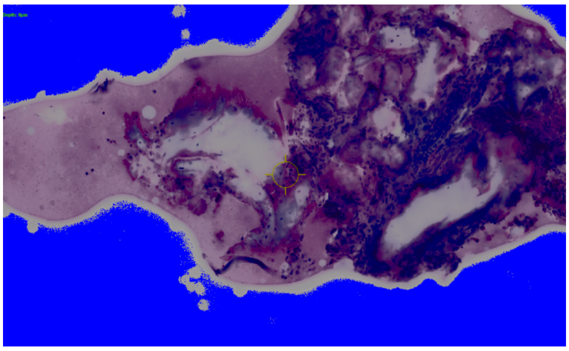

Figure S4. Case ID14. Identification of necrotic areas.

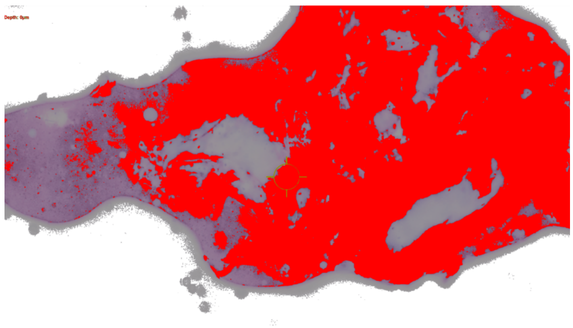

Figure S5. Case ID14. Images showed areas with inflammatory infiltrate (highlighted in red).

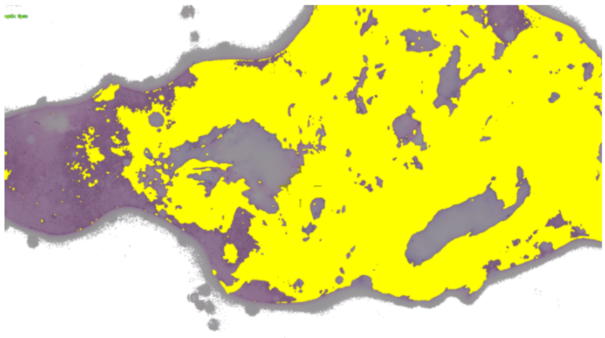

Figure S6. Case ID14. Images showed nuclei highlighted in yellow
